# Pathophysiology of gas exchange impairment in extreme prematurity: Insights from combining volumetric capnography and measurements of ventilation/perfusion ratio

**DOI:** 10.3389/fped.2023.1094855

**Published:** 2023-03-15

**Authors:** Theodore Dassios, Emma E. Williams, J. Gareth Jones, Anne Greenough

**Affiliations:** ^1^ Women and Children’s Health, School of Life Course Sciences, Faculty of Life Sciences and Medicine, King’s College London, London, United Kingdom; ^2^ Neonatal Intensive Care Unit, Patras University Hospital, Patras, Greece; ^3^ Cambridge University Clinical School, Cambridge, United Kingdom; ^4^ National Institute for Health Research (NIHR) Biomedical Research Centre Based at Guy’s and St Thomas’ NHS Foundation Trust and King’s College London, London, United Kingdom

**Keywords:** extreme prematurity, ventilation to perfusion ratio, right to left shunt, volumetric capnography, gas exchange

## Abstract

**Background:**

Infants born extremely preterm often suffer from respiratory disease and are invasively ventilated. We aimed to test the hypothesis that gas exchange in ventilated extremely preterm infants occurs both at the level of the alveoli and *via* mixing of fresh deadspace gas in the airways.

**Methods:**

We measured the normalised slopes of phase II and phase III of volumetric capnography and related them with non-invasive measurements of ventilation to perfusion ratio (V_A_/Q) and right-to-left shunt in ventilated extremely preterm infants studied at one week of life. Cardiac right-to-left shunt was excluded by concurrent echocardiography.

**Results:**

We studied 25 infants (15 male) with a median (range) gestational age of 26.0 (22.9–27.9) weeks and birth weight of 795 (515–1,165) grams. The median (IQR) V_A_/Q was 0.52 (0.46–0.56) and shunt was 8 (2–13) %. The median (IQR) normalised slope of phase II was 99.6 (82.7–116.1) mmHg and of phase III was 24.6 (16.9–35.0) mmHg. The V_A_/Q was significantly related to the normalised slope of phase III (*ρ* = −0.573, *p* = 0.016) but not to the slope of phase II (*ρ* = 0.045, *p* = 0.770). The right-to-left shunt was not independently associated with either the slope of phase II or the slope of phase III after adjusting for confounding parameters.

**Conclusions:**

Abnormal gas exchange in ventilated extremely preterm infants was associated with lung disease at the alveolar level. Abnormal gas exchange at the level of the airways was not associated with quantified indices of gas exchange impairment.

## 1. Introduction

Respiratory disease is a common complication of extremely preterm birth (occurring before 28 completed weeks of gestation). In a recent five year whole-population study we reported that nine in ten extremely preterm infants in England required surfactant replacement therapy shortly after birth and 58% developed moderate or severe bronchopulmonary dysplasia (1). Despite the increased use of non-invasive ventilation and less invasive surfactant administration (2), invasive mechanical ventilation is very common in extreme prematurity, with 95% of infants requiring invasive ventilation for a median duration of 10 days ranging up to several months in the most severe cases (1).

Conventional respiratory physiology describes that gas exchange takes place at the level of the alveolar membrane where the gas in the alveoli meets the capillary network (3). The respiratory gases are actively transported to the alveoli by inspiration or inflation (3) and cross the alveolar membrane by passive diffusion (4). This theory has recently been challenged by bench and clinical studies that reported that effective carbon dioxide elimination is possible with tidal volumes smaller that the dead space, possibly *via* spikes of fresh gas which penetrate through the dead space and create an interface of gas exchange in the conducting airways (5–7).

Accepting that gas exchange can occur with tidal volumes smaller than the dead space has practical implications in setting the appropriate targeted tidal volume during volume targeted ventilation, as too low tidal volumes have been associated with atelectasis, inefficient ventilation and lung inflammation (8) and too high tidal volumes with overdistention and iatrogenic lung injury (9).

We have previously used a novel methodology to non-invasively calculate indices of gas exchange impairment such as the ventilation to perfusion ratio (V_A_/Q) and right-to-left shunt in premature infants with BPD (10), in ventilated infants with pulmonary interstitial emphysema (11) and healthy term infants without respiratory disease (12, 13). We have also used latest generation, low-dead space, mainstream volumetric capnography to measure the anatomical and alveolar dead space in ventilated premature infants with respiratory distress syndrome or bronchopulmonary dysplasia (14). Volumetric capnography can differentiate the phases of gas emptying from the lungs: emptying of mixed gas from the conducting airways and alveoli (phase II) or purely from the alveoli (phase III) with steeper phases representing more severe ventilation inhomogeneity (15, 16). These phases can thus be used to discriminate the origin of the expired gas from either the airways or purely from the alveoli. Combining concurrent measurements of volumetric capnography with non-invasive measurements of V_A_/Q and shunt could thus give us valuable insight into the location of gas exchange impairment, i.e., whether gas exchange happens solely at the alveolar membrane or in the conducting airways also. A significant association of a low V_A_/Q with an increased slope of phase II would suggest that abnormal gas mixing does indeed occur in the airways. If, however, abnormalities of V_A_/Q only correlate with the slope of phase III, this would point towards the traditional dogma that abnormal gas exchange happens predominantly at the level of the alveolar membrane and not in the airways.

We hypothesised that in ventilated extremely preterm infants, V_A_/Q and shunt would be related with the slopes of phase II and III of volumetric capnography, signifying that abnormal gas exchange in these infants originates both from abnormal mixing of gas from the airways as well as from the alveoli. Our aim was to test this hypothesis.

## 2. Materials and methods

### 2.1. Subjects

This was a secondary analysis of a study that aimed to non-invasively estimate the alveolar surface area in preterm infants [registered on clinicaltrials.gov: NCT 04936477]. Extremely preterm (born <28 completed weeks of gestation), ventilated infants were recruited between 01/10/2020 and 31/01/2022 at the Neonatal Intensive Care Unit, King's College Hospital NHS Foundation Trust, London UK. Recruited infants were studied at one week after birth as this time point has been found to be predictive of a later development of respiratory morbidity (17). Infants were ventilated by volume-targeted ventilation using the SLE 6,000 neonatal ventilator (SLE, Croydon, UK) and were intubated with Cole's shouldered endotracheal tubes which minimise expiratory leak (18). The original study was approved by the Brighton and Sussex Research Ethics Committee, UK [REC 20/PR/0299] and eligible infants were recruited after written informed parental consent.

### 2.2. Ventilation/perfusion ratio and right-to-left shunt

The relative position of an individual's SpO_2_ vs. FiO_2_ curve against the oxyhaemoglobin dissociation curve (ODC) can be used to calculate the degree of right-to-left shunt and ventilation/perfusion (V_A_/Q) inequality as a non-invasive measure of oxygenation impairment (19–21). Right-to-left shunt causes a decrease in arterial oxygen saturation which cannot be corrected by increasing the level of inspired oxygen. The level of right-to-left shunt can be thus quantified by the degree of the depression of the SpO_2_ vs. FiO_2_ curve against the ODC curve. Contrarily, a reduction in the ventilation to perfusion ratio (V_A_/Q) results in reduced oxygen content in post-alveolar blood which produces a rightward shift of the curve which can be overcome by increasing the fraction of inspired oxygen. Thus, the reduction in V_A_/Q can be quantified by the degree of the right shift of the curve (20). The shift is derived from the gradient between the ODC and the individual subject curve measured on the steep part near 75% of the SpO_2_. The magnitude of the right shift of the curve is proportional to the reduction in V_A_/Q. All infants were nursed supine for consistency, as regional pulmonary blood flow and the delivery of ventilation to different zones of the lungs can vary depending on position (22). To calculate the V_A_/Q three to five paired measurements of transcutaneous oxygen saturation (SpO_2_) and fraction of inspired oxygen (FiO_2_) were recorded by altering the provided FiO_2_ so that the SpO_2_ varied within a predefined range of 86%–100%. A previously published computer software was used (23, 24). The computer software superimposed a best-fit ODC to individual infant data, thus giving a calculated value of V_A_/Q (20). The software used the fetal ODC as reference and incorporated the concurrent haemoglobin value (mg/dl) measured by blood gas analysis. The partial pressure of oxygen and CO_2_ by arterial blood gas analysis was also recorded.

### 2.3. Volumetric capnography

Flow and expired carbon dioxide (CO_2_) were recorded with an NM3 respiratory profile monitor (Philips, Respironics) with customised Spectra software that displayed flow, volume, airway pressure and CO_2_ over time (Grove Medical, UK). The NM3 monitor has a combined flow, pressure, and mainstream CO_2_ sensor (Capnostat-5) with a dead space of 0.8 ml and a CO_2_ response time of less than 60 ms. The NM3 combined sensor was placed between the ventilator circuit and the endotracheal tube to collect expired CO_2_ and volume data which were used for the construction of volumetric capnograms. The flow signal was integrated over time to calculate the expired tidal volume. The expiratory time (ms) was measured starting from the start of negative flow up to where the flow equals zero.

**Figure 1:**
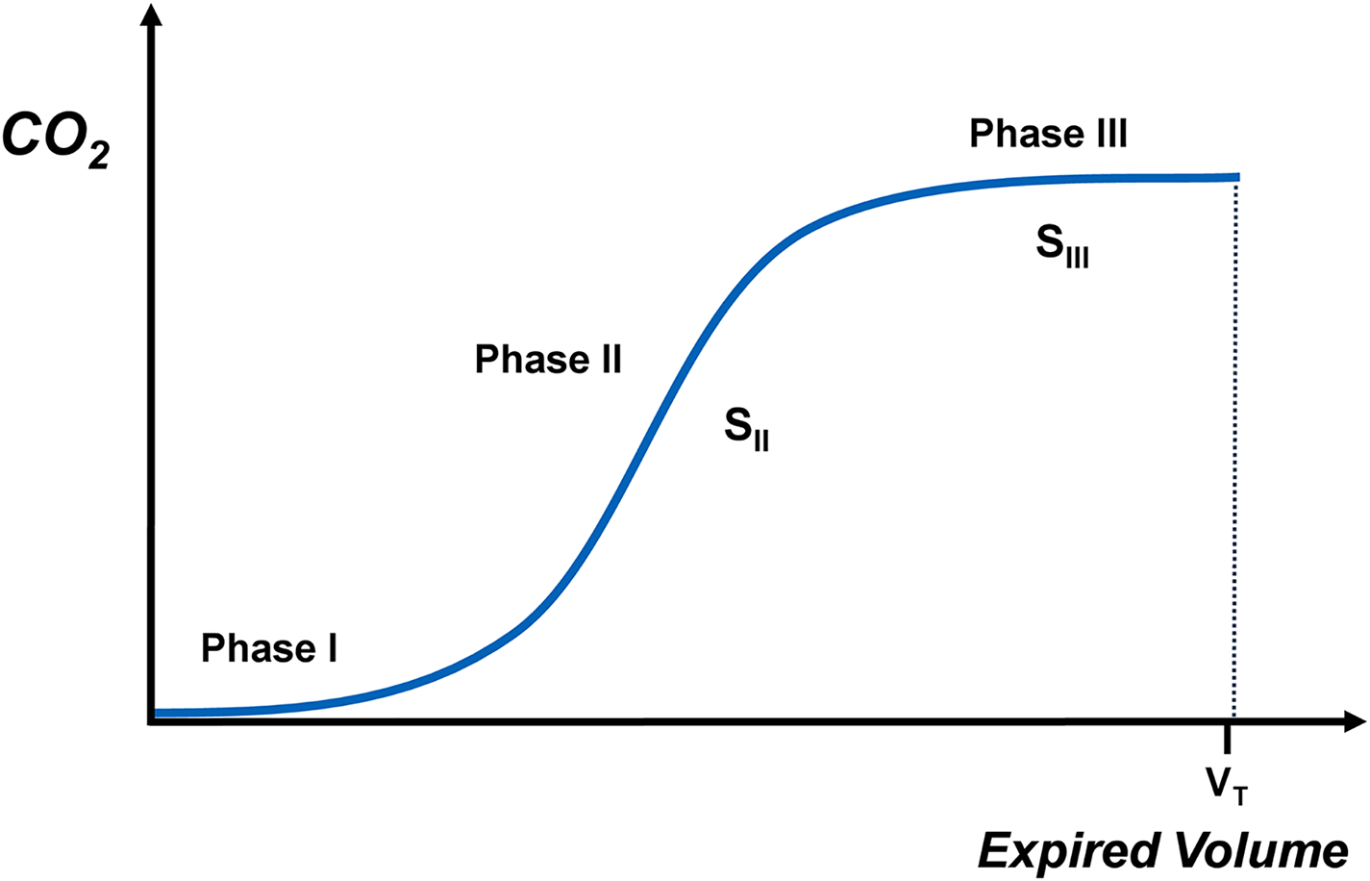
Schematic depiction of the phases of volumetric capnography and the slopes of phase II (S_II_) and III (S_III_).

Data were extracted from the Spectra software with a sampling rate of 100 Hz. Ten volumetric capnograms were constructed for each infant. The volumetric capnogram was split into three phases: phase I, phase II and phase III (Figure 1) (15, 16). Phase I represents the initial part of expiration when gas was expired from the large conducting, non-gas exchanging airways. Phase II corresponds to the middle part of expiration and contains a mixture of gas from the large airways with alveolar gas from distal airways. Phase III is the final phase of expiration when pure alveolar gas is expired resulting in an alveolar plateau. The gradient (slope) of phase II and III (S_II_ and S_III_ respectively) were calculated by fitting a regression line to the respective phases. The shape of each capnogram was evaluated and if an alveolar plateau was present, S_III_ was calculated. The individual capnogram shape was considered rather than calculating measurements at a fixed portion of the expired tidal volume, with a minimum of ten sample points required for S_III_ (25, 26). The average values obtained from each of the ten capnograms were calculated for each infant. Values of S_II_ and S_III_ were normalised to tidal volume to account for anthropometric differences (16, 27). All evaluations were made by the same investigator (EW). The minute ventilation was also calculated as the product of the generated expiratory tidal volume times the observed respiratory rate.

### 2.4. Cardiac measurements

Targeted echocardiography (ECHO) was concurrently performed by a certified neonatal clinician utilising the Philips Affiniti 50G ultrasound system (Philips, Amsterdam, NL) and reviewed by an external Consultant in Paediatric Cardiology. Since the non-invasive measurement of right-to-left shunt cannot differentiate the origin of the shunt as intrapulmonary or cardiac, echocardiography was performed to confirm that cardiac shunting was predominantly left-to-right and to exclude any right-to-left shunting at the level of the ductus arteriosus. The following parameters were measured: presence and direction of any intracardiac shunting, any patent ductus arteriosus (PDA) (and if present the size and direction of flow) and pulmonary flow (Qp) (28).

### 2.5. Information from the medical records and nursing records

The following parameters were collected from the medical notes: full course of antenatal corticosteroids (at least two doses) (yes/no), sex, gestational age (weeks), birth weight (kg), birth weight z-score (29), Apgar score at 10 min, total days invasively ventilated, administration of surfactant, PDA medically treated or surgically ligated (30), intraventricular haemorrhage (IVH) grade III or IV (yes/no) (31), bronchopulmonary dysplasia at 36 week postmenstrualage (yes/no) (32), retinopathy of prematurity requiring laser photocoagulation (33), discharge on home oxygen (yes/no), postmenstrual age at discharge (weeks), weight at discharge (kg).

### 2.6. Statistical analysis

The data were tested for normality using the Kolmogorov-Smirnov test and found to be non-normally distributed. Data were presented as median and range. The main aim of the analysis was to describe the relationship of the V_A_/Q and right-to-left shunt with S_II_ and S_III_, but the relationships of V_A_/Q and shunt were also tested according to the possible confounding parameters of sex, antenatal steroids, gestational age and birth weight z-score. Spearman's ρ correlation analysis was utilised to assess the strength of the relationships between the V_A_/Q and right-to-left shunt with the S_II_, S_III_, gestational age and birth weight z-score. The Mann Whitney *U* Test was performed to determine if differences in V_A_/Q and shunt were statistically significant in male vs. female infants and infants that received a full course of antenatal steroids vs. the ones that did not. The relationship of the V_A_/Q with the S_III_ was graphically depicted with linear regression analysis. The level of statistical significance was set at *p* = 0.05. To examine the independent association of factors that were significantly related to right-to-left shunt, multivariable linear logistic regression analysis was performed, with the right-to-left shunt as dependent variable. To determine which parameters were inserted into the multivariable linear regression model, the level of statistical significance was set at *p* = 0.1. Multi-collinearity among the independent variables in the regression analysis was assessed by examination of a correlation matrix for the independent variables. Statistical analysis was performed using SPSS software version 27 (SPSS Inc., Chicago IL).

## 3. Results

Twenty five infants (15 male) were included with a median (range) gestational age of 26.0 (22.9–27.9) weeks, a median (range) birth weight of 795 (515–1,165) grams and a birth weight z-score of −0.25 (−2.16–0.99) and were studied at a postnatal age of 7 (5–9) days. Nineteen infants had a complete course of antenatal corticosteroids and all infants received at least one dose of surfactant. The targeted tidal volume during mechanical ventilation was 6.0 (5.0–6.8) ml/kg and the mean airway pressure was 10.9 (7.2–13.6) cmH_2_O. Fifteen infants were medically treated for a patent ductus arteriosus and two infants had ligation of the PDA. Five infants were diagnosed with a grade III/IV intraventricular haemorrhage. Two infants required surgical intervention for necrotising enterocolitis and four infants required treatment for retinopathy. Further demographics and clinical outcomes of the study population are presented in Table 1. The median (interquartile range) partial pressure of oxygen calculated by arterial blood gas analysis was 60 (38–73) mmHg and of CO_2_ was 45 (39–53) mmHg. The median (interquartile range) minute ventilation was 220 (192–328) ml/kg/min.

**Table 1:** Characteristics of the study population.

*N *= 25	
Antenatal corticosteroids	18 (72)
Male sex	15 (60)
Gestational age (weeks)	26.0 (22.9–27.9)
Birth weight (grams)	795 (515–1,165)
Birth weight z score	−0.25 (−2.16–0.99)
Day of life at study	7 (5–9)
Postmenstrual age at study (weeks)	26.7 (23.4–28.9)
Weight at study (grams)	785 (590–1,155)
PDA treatment (mm)	17 (68)
Pulmonary perfusion (ml/min/kg)	394 (192–1,029)
Intraventricular haemorrhage	5 (20)
Total duration of mechanical ventilation (days)	46 (8–153)
Surgical intervention for necrotising enterocolitis	2 (8)
Treatment for retinopathy	4 (16)
Postmenstrual age at discharge (weeks)	44.1 (37.9–53.4)
Weight at discharge (grams)	3,840 (2,170–5,980)
Survival to discharge	23 (92)
Severe bronchopulmonary dysplasia or death	17 (68)
Home oxygen requirement	16 (64)

Median (range) or number (%).

All constructed volumetric capnograms had adequate definition of the phase II for the calculation of S_II_. The construction of volumetric capnograms with a phase III that was of adequate quality to calculate the S_III_ was feasible in 17 of the 25 included infants. The median (IQR) V_A_/Q and shunt were not different in the infants in whom the calculation of S_III_ was feasible [0.48 (0.45–0.56) and 5 (2–11) %] vs. the ones where calculation of S_III_ was not feasible [0.53 (0.49–0.57), *p* = 0.069 and 11 (2–16) %, *p* = 0.463 respectively].

**Figure 2:**
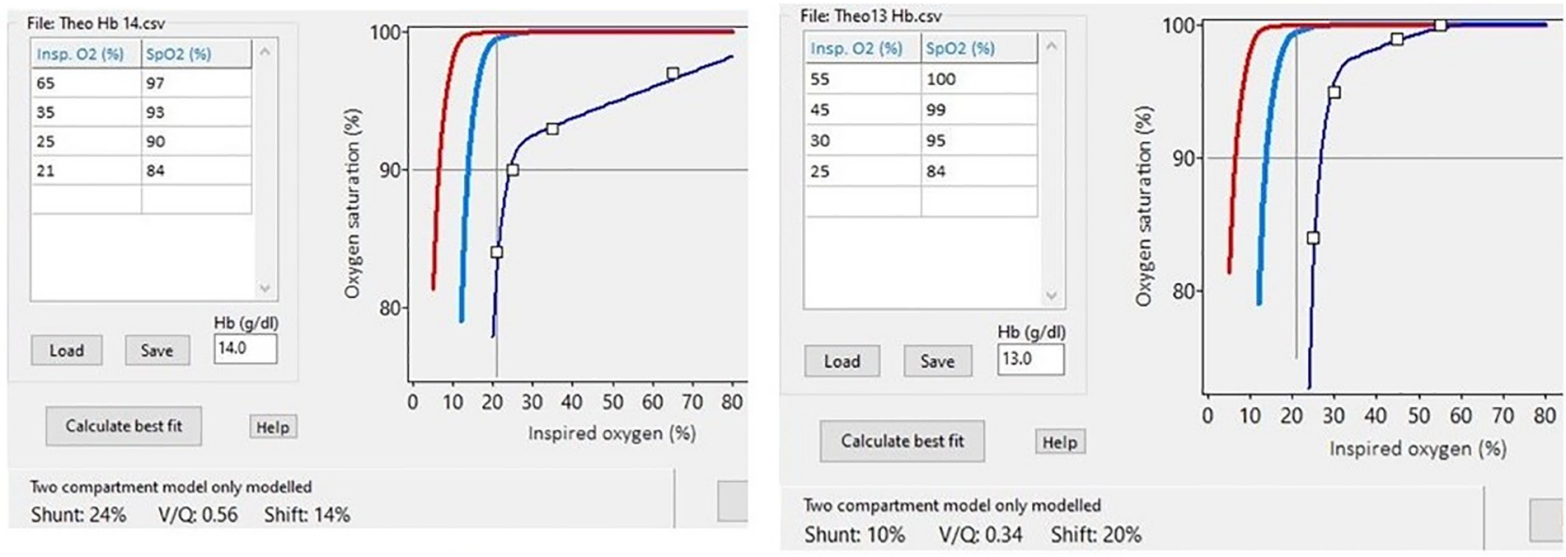
Representative examples of using the SpO_2_ vs. FiO_2_ curve against the oxyhaemoglobin dissociation curve (red line) to measure various degrees of abnormalities of the V_A_/Q and right-to-left shunt.

**Figure 3:**
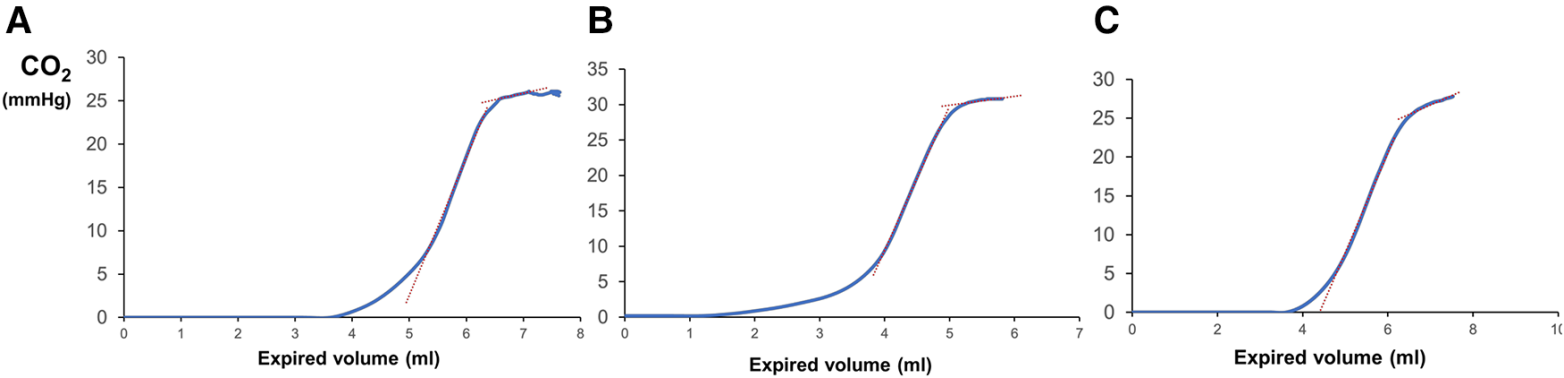
Volumetric capnograms with the corresponding values of the expired tidal volume and expired CO_2_. Please note the steeper slope of phase III in capnogram “c” compared to “a” and “b” and the overall high dead space to expired tidal volume ratios with a prolonged phase I.

The median (IQR) V_A_/Q was 0.52 (0.46–0.56) and right-to-left shunt was 8 (2–13) %. Representative curves with low V_A_/Q and high right-to-left shunt are presented in Figure 2. The median (IQR) S_II_ was 16.6 (14.1–20.9) mmHg/ml and normalised S_II_ was 99.6 (82.7–116.1) mmHg. The median (IQR) S_III_ was 3.9 (2.6–6.4) mmHg/ml and normalised S_III_ was 24.6 (16.9–35.0) mmHg. Representative examples of volumetric capnograms are presented in Figure 3.

**Figure 4:**
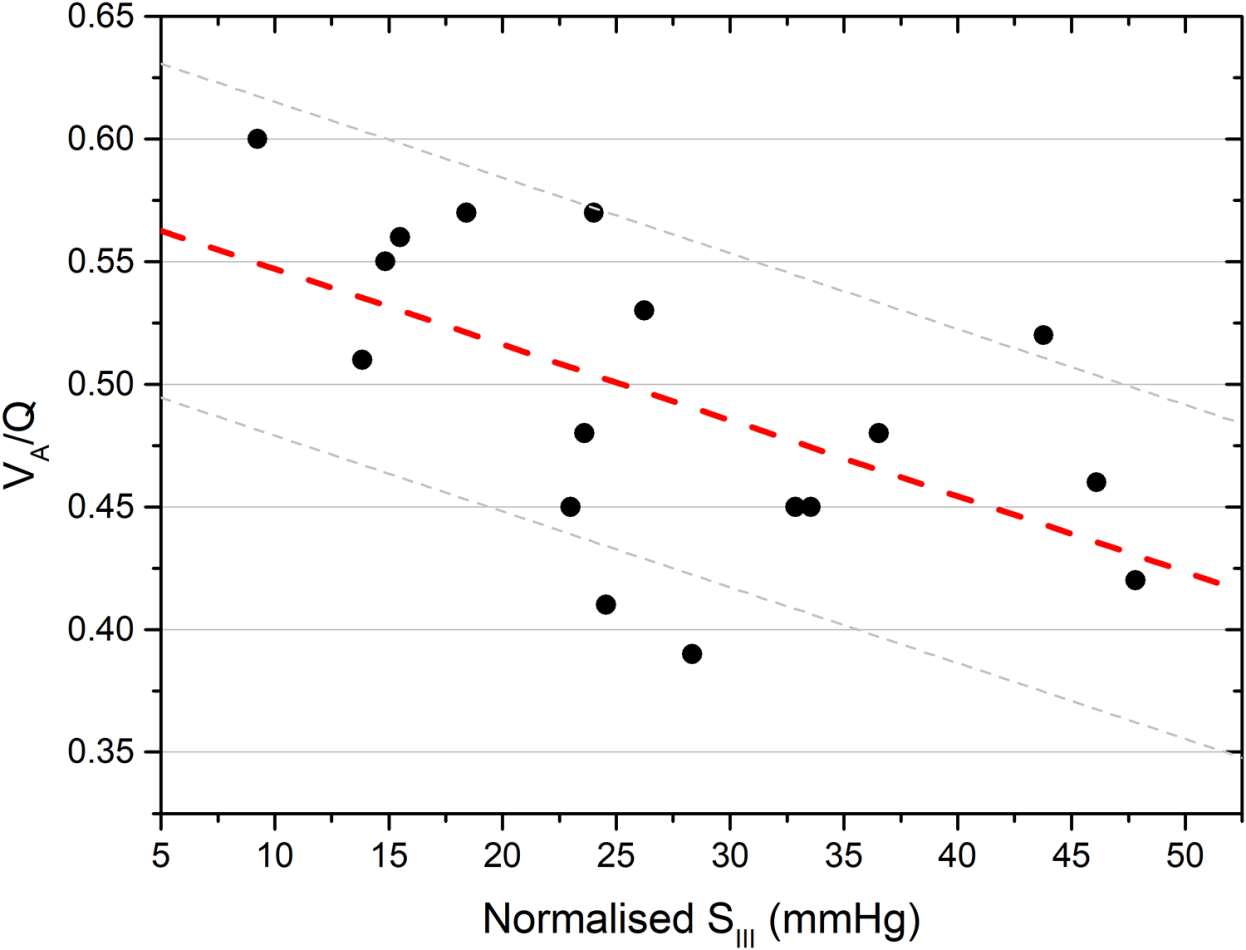
Linear regression analysis of the ventilation to perfusion ratio (V_A_/Q) and the slope of phase III of volumetric capnography (S_III_). The regression line and the 95% confidence intervals are depicted [*R*^2 ^= 0.321. *p* = 0.018].

The V_A_/Q and shunt were not statistically different in male vs. female infants (*p* = 0.808 and *p* = 0.982 for V_A_/Q and shunt respectively) or in infants that received a full course of antenatal steroids vs. the ones that did not (*p* = 0.857 and *p* = 0.286 respectively). The correlation coefficients of the univariable relationships of the V_A_/Q and right-to-left shunt with the S_II_, S_III_ and possible confounders are presented in Table 2. The V_A_/Q was significantly related to the S_III_ and the normalised S_III_ (Figure 4) but not to the S_II_, the normalised S_II_, the gestational age, the birth weight z-score, the mean airway pressure and pulmonary perfusion. The right-to-left shunt was significantly related to the gestational age and the S_II_ but not to the normalised S_II_, the S_III_, normalised S_III_, birth weight z-score, mean airway pressure and pulmonary perfusion.

**Table 2:** Univariable relationships of the V_A_/Q and shunt.

	V_A_/Q	Shunt
S_II_	*ρ* = 0.045, *p* = 0.770	*ρ* ** = 0.444, *p* = 0.026**
Normalised S_II_	*ρ* = 0.064, *p* = 0.761	*ρ* = 0.023, *p* = 0.914
S_III_	*ρ* ** = −0.572, *p* = 0.016**	*ρ* = 0.377, *p* = 0.135
Normalised S_III_	*ρ* ** = −0.573, *p* = 0.016**	*ρ* = 0.284, *p* = 0.270
Gestational age	*ρ* = 0.051, *p* = 0.792	*ρ* ** = −0.449, *p* = 0.014**
Birth weight z-score	*ρ* = 0.096, *p* = 0.626	*ρ* = −0.332, *p* = 0.084
Mean airway pressure	*ρ* = −0.056, *p* = 0.783	*ρ* = 0.199, *p* = 0.319
Pulmonary perfusion	*ρ* = 0.093, *p* = 0.637	*ρ* = −0.177, *p* = 0.368

Significant (p<0.05) values in bold.

Following multivariable logistic regression, the right-to-left shunt was independently associated with the gestational age (adjusted *p* = 0.008, standardised coefficient = −0.51, 95% confidence intervals = −4.9 to −0.8) but not with the S_II_ (adjusted *p* = 0.159) or the birth weight z-score (adjusted *p* = 0.280).

## 4. Discussion

We demonstrated that in ventilated extremely preterm infants, impairment of gas exchange was significantly related to the final phase of gas emptying from the alveoli and not to the phase of mixed gas emptying from the airways. The second phase of volumetric capnography which described mixed gas emptying from the conducting airways and alveoli was not independently related either to the ventilation to perfusion ratio or to the right-to-left shunt.

The hypothesis that phase II would be associated with impaired oxygenation was not verified in our study and our results point towards the conclusion that conventional respiratory physiology remains valid as gas exchange and oxygenation impairment likely occur predominantly at the level of the alveolar membrane. This conclusion is not in agreement with some previous studies that have suggested that alveolar ventilation can occur at tidal volumes below the dead space, possibly *via* mixing of fresh and dead space gas in the conducting airways in infants ventilated with high rates and very short time constants in a mechanism which is similar to high frequency oscillation (34). Although this theory was supported by well conducted bench studies (5, 6) the main clinical study behind it, was conducted by estimating the dead space by adding instrumental dead space measured by water displacement to a standard value of 0.5 ml/kg for the dead space of the distal trachea and the main bronchi; a value which was not backed in the original paper with cited evidence (7). Although not specifically clarified, the tidal volume appears to have been measured by the inbuilt flow sensor of the used ventilator which has a dead space of 1.7 ml and an accuracy of ±0.5 ml according to the manufacturer. These technical characteristics could account for some of the observed differences. We have previously used a flow sensor with a low dead space of 0.8 ml to measure a dead space of approximately 5 ml/kg in preterm infants with respiratory distress syndrome and bronchopulmonary dysplasia and reported that the dead space was large but the tidal volume was higher than the dead space with a median dead space to tidal volume ratio of approximately 0.75 (14). In the same paper we described that in response to this inefficient ratio, ventilated preterm infants possibly increase their spontaneous respiratory rate to maintain alveolar ventilation (33).

It is interesting to note that the debate on whether efficient ventilation can occur with tidal volumes that are lower than the dead space refers predominantly to the extremely preterm infants as the contribution of the fixed amount of the instrumental dead space is relatively higher in the smallest infants, which are the ones that would benefit more from lung-protective ventilation strategies. A very low targeted tidal volume though, is not always lung protective, as too small volumes have been associated with an increased work of breathing (35), a higher incidence of extubation failure (36) and a pro-inflammatory state (8). A potential practical recommendation that emerges from our results is that the targeted tidal volume in ventilated preterm infants should not be lower than the total anatomical, alveolar and instrumental dead space and very low targeted tidal volumes (below 5 ml/kg) should be avoided.

Studies of ventilation in extreme prematurity frequently report a large range of measured values of tidal volumes or other ventilator parameters such as the oxygen or mean airway pressure requirement (37). While our study supports traditional respiratory physiology, i.e., that gas exchange would require a targeted tidal volume which exceeds the dead space, some differences between our results and previous studies (5, 7) might be explained by population characteristics and some expected variation within any population. For example, in our study we report a range of tidal volume of 4–8 ml/kg while all included infants had a tightly controlled, “normal” arterial carbon dioxide. This implies that not all infants require the same volume targeting and that further metabolic and respiratory parameters might influence the rate of carbon dioxide elimination and the corresponding tidal volume requirement. In this sense, the most sensible approach might be an individualised strategy that provides adequate carbon dioxide control, while avoiding excessive values of low or high tidal volumes but also avoids excessively high respiratory rates that might represent an endogenous effort of the infants to maintain alveolar ventilation in the face of an inefficiently low targeted tidal volume. We suggest that the target parameter for such an approach would be the end tidal carbon dioxide, since it can be continuously monitored and this is the parameter whose fluctuations have been associated with intracranial haemorrhage in preterm infants (Tamura K, Williams EE, Dassios T, Pahuja A, Hunt KA, Murthy V, Bhat P, Bhat R, Milner A, Greenough A. End-tidal carbon dioxide levels during resuscitation and carbon dioxide levels in the immediate neonatal period and intraventricular haemorrhage. Eur J Pediatr. 2020 Apr; 179(4): 555–559.).

Our study has strengths and some limitations. We used a novel combination of three non-invasive methodologies (oxyhaemoglobin dissociation curve, volumetric capnography, echocardiography) to describe a complex phenomenon with significant clinical implications. We included infants of extremely premature gestation as low as 22 weeks of gestation which is a vulnerable and rare population. Having only included infants of less than 28 weeks of gestation we could not describe how a lower gestation or birth weight would affect the slopes of volumetric capnography or the gas exchange impairment indices, but we have previously separately described abnormal V_A_/Q and shunt in preterm infants and steeper volumetric capnography slopes in preterm infants compared to their term counterparts reflecting a developmental/maturation effect (16, 27). Our study was a secondary analysis of a pre-existing dataset that was collected as part of a prospective study, so some element of methodological bias cannot be excluded. Since, however, this was a secondary analysis the selection of the included infants was not influenced by the design of this specific study.

In conclusion, we reported that abnormal gas exchange in ventilated extremely preterm infants was associated with lung disease at the alveolar level and that abnormal gas exchange at the level of the airways was not associated with quantified indices of gas exchange impairment such as the ventilation to perfusion ratio and right-to-left intrapulmonary shunt.

## Data Availability

The original contributions presented in the study are included in the article, further inquiries can be directed to the corresponding author.
